# CREB1-driven expression of miR-320a promotes mitophagy by down-regulating VDAC1 expression during serum starvation in cervical cancer cells

**DOI:** 10.18632/oncotarget.5318

**Published:** 2015-10-14

**Authors:** Qin-qin Li, Le Zhang, Hai-ying Wan, Min Liu, Xin Li, Hua Tang

**Affiliations:** ^1^ Tianjin Life Science Research Center and School of Basic Medical Sciences, Tianjin Medical University, Tianjin, China

**Keywords:** CREB1, miR-320a, mitophagy, serum starvation, VDAC1

## Abstract

The altered expression of miRNAs in response to stresses contributes to cancer pathogenesis. However, little is known regarding the mechanism by which cellular stresses drive alterations in miRNA expression. Here, we found that serum starvation enhanced mitophagy by downregulating the mitophagy-associated protein voltage-dependent anion channel 1 (VDAC1) and by inducing the expression of miR-320a and the transcription factor cAMP responsive element binding protein 1(CREB1). Furthermore, we cloned the promoter of miR-320a and identified the core promoter of miR-320a in the upstream −16 to −130 region of pre-miR-320a. Moreover, CREB1 was found to bind to the promoter of miR-320a to activate its expression and to induce mitophagy during serum starvation. Collectively, our results reveal a new mechanism underlying serum starvation-induced mitophagy in which serum starvation induces CREB1 expression, in turn activating miR-320a expression, which then down-regulates VDAC1 expression to facilitate mitophagy. These findings may provide new insights into cancer cell survival in response to environmental stresses.

## INTRODUCTION

MicroRNAs (miRNAs) are a class of noncoding RNAs that are approximately 22 nucleotides (nt) in length and that are important regulators of gene expression. miRNAs are involved in diverse physiological and pathological processes and environmental stresses, including cell proliferation [1], differentiation [1], apoptosis [2], autophagy [3], tumorigenesis [4], and even epigenetic regulation [5]. The expression of miRNAs is regulated by many factors associated with various environmental stresses, such as starvation [6], nutrition shortage [7], hypoxia, inflammation [8], oxidative stimulation, and even anti-cancer drug treatments. Emerging studies have reported that miRNA expression is altered under these stresses through crosstalk with the tumor microenvironment. In response to stress, cancer cells often undergo changes in gene expression to facilitate their survival [7]. For malignant tumors, the blood supply is relatively insufficient, thereby subjecting the tumor tissues to an ischemic environment. In this study, we screened the differential expression profiles of miRNAs during serum starvation in the HeLa cervical cancer cell line by miRNA microarray, and we chose miR-320a for further studies because its expression changed the most.

Previous studies have revealed that miR-320a is associated with the proliferation of cancer cells. miR-320a inhibits the proliferation of granulosa [9] and leukemic cells [10]; however, Kim et al. reported that miR-320a promotes proliferation in Dgcr8-deficient embryonic stem cells [11]. Other roles of miR-320a in diverse physiological and pathological processes have been discovered more recently. miR-320a expression is regulated by hypoxia, regulates the function of vascular endothelial cells by targeting NRP1, and has the potential to be developed as an anti-angiogenic or anti-cancer drug [12]. Moreover, miR-320a is a critical component of the PTEN tumor suppressor axis that acts in stromal fibroblasts to reprogram the tumor microenvironment and to curtail tumor progression [13]. MiR-320a acts as a prognostic factor and inhibits metastasis of salivary adenoid cystic carcinoma by targeting integrin beta 3[14]. miR-320a is a tumor-suppressive miRNA in glioma, at least, partially through regulating insulin-like growth factor-1 receptor and its downstream, AKT and ERK signaling pathways [15]. Furthermore, low miR-320a expression was found to be associated with invasive breast cancer progression and predicts poor patient prognosis [16]. An anti-miR-320a oligo was found to regulate insulin resistance in adipocytes by improving insulin-PI3K signaling pathways [17]. However, the mechanism by which miR-320a expression is regulated is not clear. Additionally, the role of miR-320a in the regulation of mitophagy in cervical cancer cells has not been elucidated.

Mitophagy is a process by which dysfunctional mitochondria are selectively removed by autophagy [18–20]. During this process, damaged mitochondria are incorporated into double-membrane structures known as autophagosomes, which are then delivered to lysosomes for degradation [19]. Three principal methods are presently used to monitor the number of autophagosomes, including electron microscopy, light microscopy detection of the subcellular localization of LC3, and biochemical detection of the membrane-associated form of LC3. The most traditional method is electron microscopy. The assessment of autophagosome number by electron microscopy requires considerable specialized expertise, and is becoming increasingly replaced by light microscopic and biochemical methods that are more widely accessible to researchers in different fields [21]. Mitophagy has a crucial function in maintaining mitochondrial quality and cellular homeostasis. This study established a relationship between miR-320a and mitophagy. We found that mitophagy is caused by the decreased expression of an outer mitochondrial membrane protein known as voltage-dependent anion channel 1 (VDAC1).

VDAC is a 31 kDa pore-forming protein found in all eukaryotes. This protein functions as a gatekeeper for the entry and exit of mitochondrial metabolites, thereby controlling the crosstalk between mitochondria and the rest of the cell. Three versions of VDAC have been identified: VDAC1, VDAC2 and VDAC3 [22]. VDAC1 is the most abundant isoform in most cells, being 10 times more prevalent than VDAC2 and 100 times more prevalent than VDAC3 in HeLa cells [23].

Here, we revealed that serum starvation induced mitophagy, altered the miRNA expression profile and increased cAMP responsive element binding protein 1 (CREB1) expression in cervical cancer cells. More importantly, we found that miR-320a facilitated mitophagy by downregulating VDAC1 expression. Furthermore, we characterized the promoter of miR-320a and determined that the upregulation of CREB1-activated miR-320a expression induced mitophagy in serum-starved HeLa and C33A cells. Taken together, our results demonstrated that serum starvation induced CREB1 expression to activate miR-320a expression, which then suppressed VDAC1 expression to promote mitophagy, enhancing the survival of cervical cancer cells. The clarification of these mechanisms provides new insight into the response of cancer cells to environmental stresses.

## RESULTS

### Serum starvation promotes mitophagy in HeLa cells

**Figure 1:**
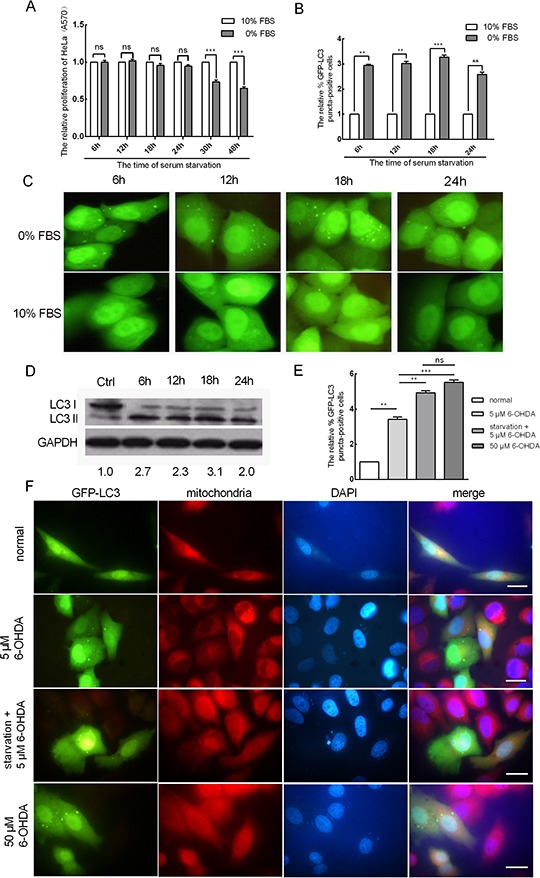
Serum starvation promotes mitophagy in HeLa cells **A.** The relative proliferation of HeLa cells after serum starvation for 6, 12, 18, 24, 30 and 48 h as determined by MTT assay (A570). **B.** Serum starvation induces EGFP-LC3 translocation. HeLa cells were serum starved for 6, 12, 18 and 24 h after being transfected with EGFP-LC3 for 24 h. The percentage of EGFP-LC3 puncta-positive cells was quantified using a threshold of ≥ 5 dots per cell. **C.** Representative images from the quantification shown in (B) Magnification, 100 ×. **D.** The expression levels of LC3-I and LC3-II in HeLa cells when serum starved for 6, 12, 18 and 24 h as determined by western blot analysis. **E.** The HeLa cells were treated as indicated. The nucleus and mitochondria were stained by DAPI (blue) and MitoTracker (red). **F.** Representative images from the quantification shown in (E) Magnification, 100 ×; scale bar, 20 μm. The error bars are ± SEM. **p < 0.01, ***p < 0.001.

Previous evidence has shown that serum starvation induces autophagy [24, 25] and that long-term serum starvation leads to cell apoptosis. Here, we tested cell proliferation when HeLa cells were subjected to serum starvation for 6, 12, 18, 24, 30 and 48 h by MTT (3-(4,5-dimethylthiazol-2-yl)-2, 5-diphenyltetrazolium bromide) assays. Compared to controls, the cell proliferation of serum-starved cells was unchanged after 6, 12, 18 and 24 h. However, serum starvation for 30 and 48 h led to decreased cell proliferation (Figure 1A). This demonstrated that the early stages of serum starvation do not suppress cell proliferation. We speculated that autophagy may be induced by serum starvation, which helps HeLa cells to cope with the early stages of serum starvation. To confirm this prediction, we performed an EGFP reporter assay to quantify autophagosomes. Transient transfection of the autophagy marker GFP-LC3 was performed in HeLa cervical cancer cells for 24 h, and the cells were then serum starved for 6, 12, 18 and 24 h. Statistical analysis of fluorescence microscope images showed that the number of cell autophagosomes increased by approximately 2.9-, 3.0-, 3.3- and 2.6-fold at 6, 12, 18, and 24 h, respectively (Figure 1B and 1C). The accumulation of LC3-II is considered a biological marker of autophagy [26]; thus, western blot analysis was used to determine the expression levels of LC3-I and LC3-II in HeLa cells when subjected to serum starvation for 6, 12, 18 and 24 h. Compared to the control, the ratio of LC3-II to LC3-I markedly increased when the cells were serum starved for 6, 12, 18 and 24 h, and the accumulation of LC3-II increased by approximately 2.7-, 2.3-, 3.1- and 2.0-fold at the respective starvation periods (Figure 1D). To determine whether cell autophagy occurred in the mitochondria, MitoTracker Red (Sigma) was applied to co-stain with LC3-II to demonstrate mitophagy. 6-Hydroxydopamine (6-OHDA) was used to induce mitophagy. Fifty micromolar 6-OHDA was required to induce mitophagy for 24 h in HeLa cells at a normal serum concentration, whereas only 5 μM induced mitophagy in cells that were serum starved for 18 h, demonstrating that starvation plays the same role as 6-OHDA (Figure 1E and 1F). These results showed that serum starvation induced mitophagy and significantly facilitated the induction of 6-OHDA.

### miR-320a was upregulated in cervical cancer cells under serum starvation stress

**Figure 2:**
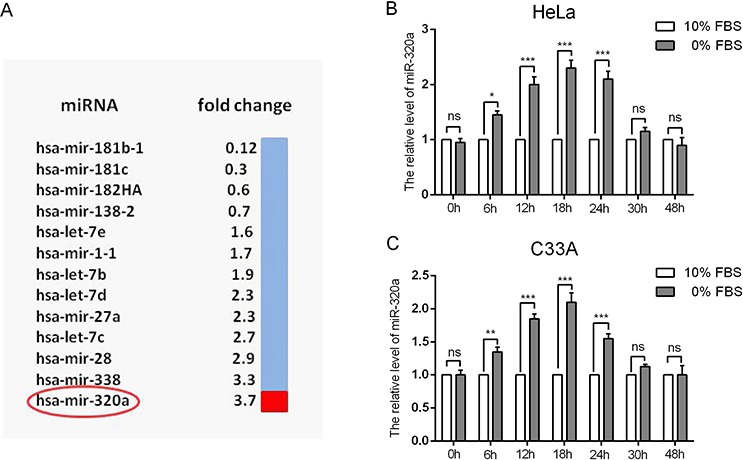
Differential expression of miRNAs and the upregulation of miR-320a in cervical cancer cells during serum starvation **A.** HeLa cells were serum starved for 48 h after reaching 60% confluence. Then, miRNA array analysis was performed. Nine miRNAs were found to be differentially expressed. **B** and **C.** Relative levels of miR-320a when HeLa and C33A cells were serum starved for 0, 6, 12, 18, 24, 30 and 48 h. The error bars are ± SEM. *p < 0.05, ***p < 0.001.

To determine the differential expression of miRNAs during serum starvation, we extracted small RNA from the HeLa cervical cancer cell line cultured in complete or serum-free culture medium to perform the miRNA microarray. In the serum-starved group, we found that 9 miRNAs (hsa-let-7b, hsa-let-7c, hsa-let-7d, hsa-let-7e, hsa-mir-1-1, hsa-mir-27a, hsa-mir-28, hsa-mir-320a and hsa-mir-338) were upregulated and that 4 miRNAs (hsa-mir-181b-1, hsa-mir-181c, hsa-mir-138-2 and hsa-mir-182HA) were downregulated compared to the non-serum-starved group (Figure 2A). Hsa-mir-320a displayed the largest difference in expression among the 9 miRNAs that were upregulated. Then, we confirmed the miRNA microarray results by qRT-PCR, which showed that serum starvation could induce the expression of miR-320a at a level similar to that of the miRNA microarray data (Figure 2B and 2C).

### miR-320a promotes mitophagy in HeLa cells

**Figure 3:**
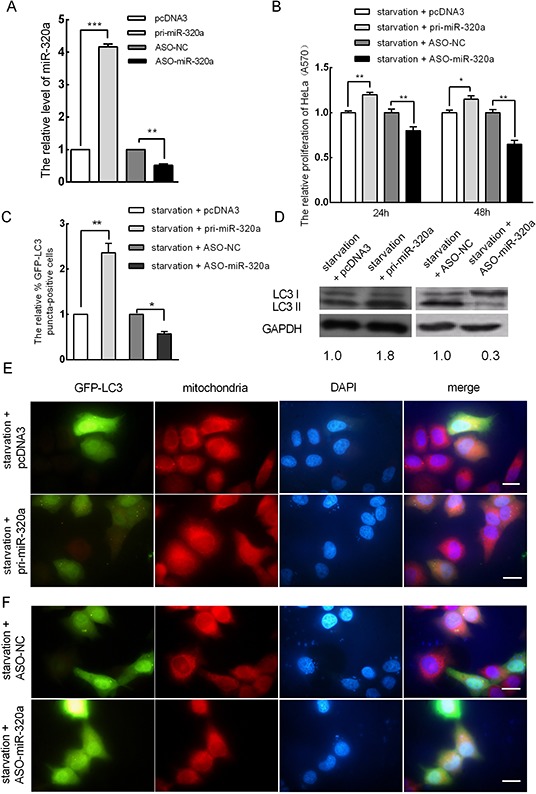
miR-320a induces mitophagy in HeLa cells **A.** The effect of the overexpression of miR-320a and ASO-miR-320a in HeLa cells. pcDNA3 and ASO-NC served as negative controls. **B.** The proliferation of HeLa cells after miR-320a inhibition and miR-320a overexpression for 24 and 48 h under starvation as determined by MTT assay (A570). **C.** The relative percentage of EGFP-LC3 puncta-positive cells when cells were transfected with pri-miR-320a or pcDNA3, ASO-miR-320a or ASO-NC and starved for 18 h post-transfection. **D.** The expression levels of LC3-I and LC3-II in HeLa cells when the cells were transfected with pri-miR-320a or pcDNA3, ASO-miR-320a or ASO-NC as determined by western blot analysis. **E.** Mitophagy in HeLa cells after miR-320a overexpression. Cells were transfected with pri-miR-320a or pcDNA3 for 24 h and then serum starved for 18 h. Magnification, 100 ×; scale bar, 20 μm. **F.** miR-320a inhibition could rescue serum starvation-mediated EGFP-LC3 translocation. Cells were transfected with ASO-miR-320a or ASO-NC for 24 h and then serum starved for 18 h. Magnification, 100 ×; scale bar, 20 μm. The error bars are ± SEM. *p < 0.05, **p < 0.01, ***p < 0.001.

We demonstrated that miR-320a is upregulated under serum starvation conditions and that serum starvation promoted mitophagy in HeLa cells. Thus, we questioned whether upregulated miR-320a expression is involved in mitophagy during serum starvation. To address this question, we generated a miR-320a expression vector (pcDNA3/pri-miR-320a) and synthesized an antisense 2-O-methoxy-modified miR-320a oligomer (ASO-miR-320a) and a scramble negative control nucleotide (ASO-NC). The efficiencies of pcDNA3/pri-miR-320a and ASO-miR-320a were confirmed by qRT-PCR, which revealed a 4.3-fold increase and a 55% decrease in miR-320a expression, respectively (Figure 3A). Similarly, to examine the effects of pri-miR-320a and ASO-miR-320a on HeLa cell proliferation under serum starvation, an MTT assay was performed. We transiently transfected the plasmids pcDNA3/pri-miR-320a and ASO-miR-320a for 24 and 48 h. Compared to the controls, miR-320a overexpression increased cell proliferation and ASO-miR-320a decreased cell proliferation (Figure 3B). Additionally, the level of mitophagy upon miR-320a overexpression increased by approximately 2.4-fold (Figure 3C and 3E) relative to the control. To further determine whether miR-320a could improve mitophagy, western blot analysis was used to detect the expression levels of LC3-I and LC3-II in HeLa cells upon miR-320a overexpression under serum starvation. Compared to the control, the accumulation of LC3-II increased by approximately 1.8-fold (Figure 3D). In addition, to determine whether elevated mitophagy upon serum starvation is due to an increased level of miR-320a, we transiently transfected ASO-miR-320a into HeLa cells to block endogenous miR-320a for 24 h, and then the transfected cells were serum starved for 18 h. Compared with the control, the inhibition of miR-320a reduced the number of autophagic cells showing GFP-LC3 dots (Figure 3C and 3F), and the accumulation of LC3-II decreased to approximately 33% of the control level (Figure 3D).

### miR-320a down-regulates VDAC1 expression to promote mitophagy in HeLa cells

**Figure 4:**
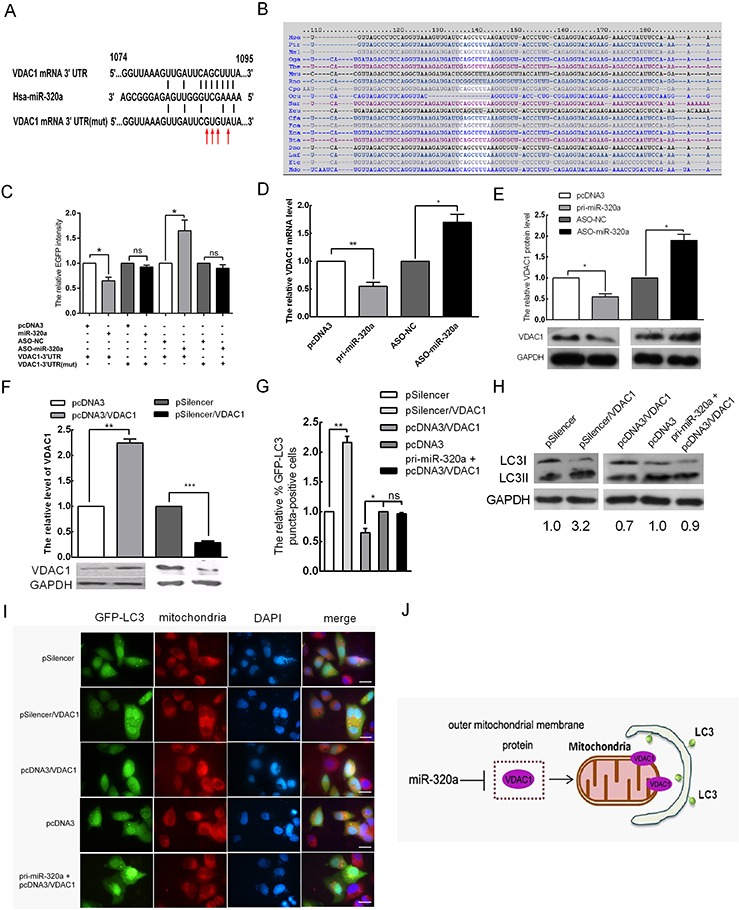
VDAC1 is a target of miR-320a that mediates the promotion of mitophagy via miR-320a in HeLa cells **A.** Predicted miR-320a binding sequence in the 3′ UTR of VDAC1 and mutation of the seed sequences. The red arrow indicates the mutated bases. **B.** The miR-320a target sites at VDAC1 3′ UTR nucleotides 1074–1095 were highly conserved across different species. **C.** The EGFP fluorescence intensity of HeLa cells decreased at 48 h after transfection with pri-miR-320a and increased following transfection with ASO-miR-320a. Mutated seed sequences abrogate the effect of miR-320a on EGFP intensity. **D** and **E.** The mRNA (D) and protein (E) levels of VDAC1 inversely correlate with miR-320a expression. **F.** The VDAC1 protein levels were examined by western blot analysis at 48 h after transfection with pcDNA3/VDAC1 or pSilencer/VDAC1. pcDNA3 and pSilencer served as the negative controls. GAPDH was used as an internal control for quantitation normalization. **G.** The relative percentage of EGFP-LC3 puncta-positive cells after transfection with pSilencer/VDAC1, pcDNA3/VDAC1 and pri-miR-320a+pcDNA3/VDAC1. pSilencer and pcDNA3 served as the negative controls. **H.** The expression levels of LC3-I and LC3-II in HeLa cells when transfected with the indicated plasmids as determined by western blot analysis. **I.** Representative images from the quantification shown in (G) Magnification, 100 ×; scale bar, 20 μm. **J.** Provisional working model of miR-320a in regulation of mitophagy. Overexpression miR-320a can reduce the level of VDAC1. Then damaged mitochondria are selectively incorporated into autophagosomes, which lead to mitophagy. The error bars are ± SEM. *p < 0.05, **p < 0.01, ***p < 0.001.

miRNAs exerts their roles by suppressing target genes [27]. To determine the target by which miR-320a enhances mitophagy in HeLa cells during serum starvation, we predicted target genes using bioinformatics tools (TargetScan, PicTar, mirnaviewer and miRCosm). miRNAs usually bind to the 3′ UTR of their targets through incomplete complementarity. VDAC1 was predicted to be a candidate target gene by all four algorithms; the binding sites of miR-320a are shown in Figure 4A and the seed sequence is highly conserved across different species (Figure 4B). VDAC1 is a mitochondrial membrane protein, and its degradation by ubiquitination is associated with mitophagy [28, 29]. To determine whether miR-320a has a direct effect on VDAC1 expression, we used an EGFP reporter assay and cloned an EGFP reporter vector containing the 3′ UTR of VDAC1 or a 3′ UTR mutant of VDAC1 with a mutation at the target sites of the miR-320a seed sequence. Co-transfection was performed with the miR-320a overexpression vector or ASO-miR-320a in HeLa cells, and the intensity of EGFP fluorescence was then analyzed. Compared with the control, miR-320a overexpression decreased EGFP expression by 35%, and ASO-miR-320a enhanced EGFP expression by 1.7-fold. In addition, the mutation of the miR-320a target site abolished the influence of the miR-320a overexpression vector and ASO-miR-320a on the fluorescent intensity (Figure 4C). miR-320a overexpression led to a 45% decrease in the VDAC1 mRNA level (Figure 4D) and to an approximately 55% decrease in the VDAC1 protein level (Figure 4E). Conversely, inhibiting miR-320a expression with ASO-miR-320a resulted in 1.7- and 2.0-fold increases in VDAC1 mRNA and protein levels, respectively (Figure 4D and 4E). These data indicated that miR-320a downregulates VDAC1 expression through directly binding to the 3′ UTR of VDAC1 in HeLa cells.

To further confirm the effect of miR-320a on the mitophagy of HeLa cells by VDAC1 targeting, we first tested the effectiveness vectors for VDAC1 overexpression (pcDNA3/VDAC1) and silencing (pSilencer/VDAC1) generated in our previous work by western blot, which revealed an approximate 2.3-fold increase and 70% decrease relative to the control group, respectively (Figure 4F). Then, we overexpressed and silenced VDAC1 to examine mitophagy; the number of autophagic cells showing GFP-LC3 dots decreased by approximately 35% and increased 2.2-fold relative to the control group, respectively (Figure 4G and 4I). To further validate the relationship between VDAC1 and mitophagy, we detected the expression levels of LC3-I and LC3-II in HeLa cells upon silencing and overexpression of VDAC1 by western blot analysis. LC3-II accumulation increased by approximately 3.2-fold and decreased by 30% in HeLa cells relative to the control, respectively (Figure 4H). To elucidate the role of miR-320a between VDAC1 and mitophagy, we detected the number of autophagic cells showing GFP-LC3 dots and the expression levels of LC3-I and LC3-II in HeLa cells co-transfected with pcDNA3/miR-320a and pcDNA3/VDAC1. Compared to the control, the number of autophagic cells showing GFP-LC3 dots and the expression levels of LC3-I and LC3-II were not statistically significant (Figure 4G–4I). Thus, miR-320a promoted mitophagy by downregulating the expression level of VDAC1 (Figure 4J).

### Identification and characterization of the miR-320a promoter

**Figure 5:**
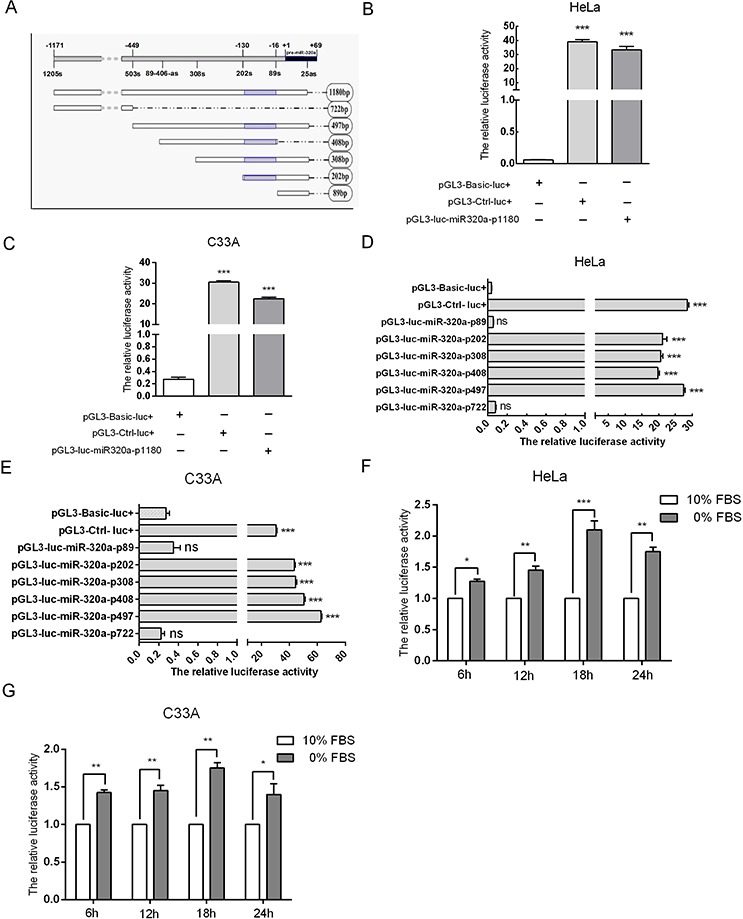
Characterization of the miR-320a promoter **A.** A diagram of the promoter fragments of miR-320a. The gray area represents the core promoter of miR-320a. **B** and **C.** The effect of the longest promoter of miR-320a in HeLa and C33A cervical cancer cells. pGL3-Ctrl-luc^+^ and pGL3-Basic-luc^+^ served as the positive and negative controls, respectively. **D** and **E.** The effects of the other promoter fragments of miR-320a on HeLa and C33A cervical cancer cells. **F** and **G.** The relative luciferase activity of miR-320a-p1180bp when HeLa and C33A cells were serum starved for 6, 12, 18 and 24 h. The error bars are ± SEM. *p < 0.05, **p < 0.01, ***p < 0.001.

To elucidate the mechanism underlying the upregulation of miR-320a during serum starvation, we predicted the promoter of miR-320a using bioinformatics and first cloned this promoter, which was named miR-320a-p1180 (Figure 5A). Next, miR-320a-p1180 was transfected into HeLa and C33A cells according to the manufacturer's instructions for a dual-luciferase reporter assay. When miR-320a-p1180 was overexpressed, luciferase activity increased by approximately 38 and 21-fold relative to the control, and this increase is almost as high as that of the positive control (Figure 5B and 5C). These data suggested that the 1180 bp fragment displayed promoter activity in HeLa and C33A cells. Furthermore, to identify the core promoter of miR-320a-p1180, the 1180 bp fragment was mutated into smaller fragments, including 722 bp, 497 bp, 408 bp, 308 bp, 202 bp and 89 bp, respectively (Figure 5A). We examined the promoter activity of the fragments, which were transfected into HeLa and C33A cells, using a dual-luciferase reporter assay and found that the 722 bp and 89 bp fragments could not express luciferase. However, the other four fragments activated luciferase expression, and all four fragments had an overlapping region at −16 to −130 bp upstream of pre-miR-320a (Figure 5A, 5D and 5E), suggesting that the core promoter of miR-320a is in the −16 to −130 sequence upstream of pre-miR-320a.

Furthermore, we tested whether the activity of the cloned miR-320a promoter could be induced by serum starvation by assaying the luciferase intensity of miR-320a-p1180. The luciferase intensity significantly increased in transfected HeLa and C33A cells at 6, 12, 18 and 24 h after serum starvation (Figure 5F and 5G), which is similar to the pattern of miR-320a expression (Figure 2B and 2C). Taken together, these results further indicated that the cloned promoter of miR-320a is responsive to short-term serum starvation.

### Upregulated CREB1 induces miR-320a expression during serum starvation

**Figure 6:**
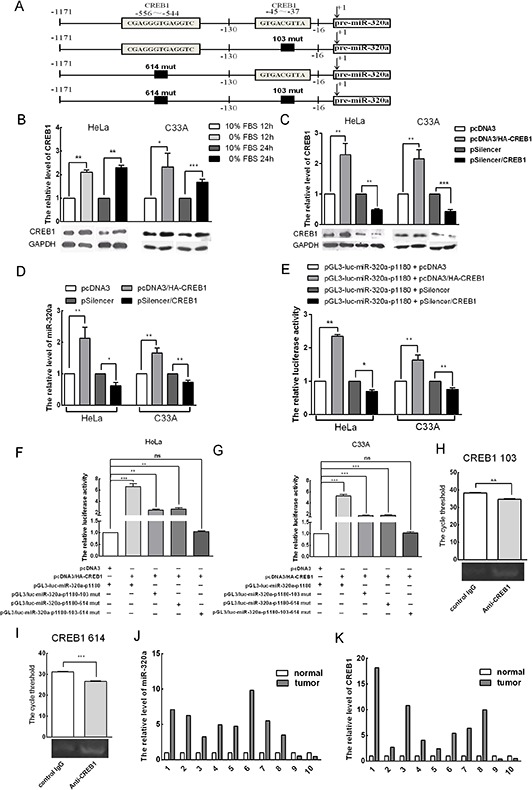
CREB1 induces miR-320a expression during serum starvation **A.** A diagram of the two CREB1 binding sites in the promoter of miR-320a and their respective mutations. **B.** Western blot analysis of the expression levels of CREB1 and GAPDH when HeLa and C33A cells were serum starved for 12 and 24 h. **C.** Effects of the overexpression and knockdown CREB1 vectors. pcDNA3/HA and pSilencer served as the negative controls. **D.** The endogenous levels of miR-320a after transfection with pcDNA3/HA-CREB1, pSilencer/CREB1 or relevant scramble controls in HeLa and C33A cervical cancer cells as determined by qRT-PCR. **E.** CREB1 regulates the promoter of miR-320a. Luciferase reporter assays were performed at 24 h after transfection with miR-320a-p1180. The *Renilla* plasmid was co-transfected as a normalization control. **F** and **G.** Plasmids were co-transfected as indicated. Then, luciferase reporter assays were performed at 24 h after transfection in HeLa and C33A cervical cancer cells. **H** and **I.** ChIP analysis reveals that CREB1 couples with the promoter of miR-320a. **J** and **K.** Expression of miR-320a and CREB1 in 10 cervical cancer tissues and adjacent non-tumor tissues as determined by qRT-PCR. The error bars are ± SEM. *p < 0.05, **p < 0.01, ***p < 0.001.

To explore how miR-320a expression is induced during serum starvation and whether transcription factors are involved in this process, we applied bioinformatics and determined that the promoter contains two CREB1 binding sites, which are in the −37 to −45 and −544 to −556 regions upstream of pre-miR-320a (Figure 6A). To investigate the relationship between CREB1 and miR-320a expression, we first analyzed the expression of CREB1 in HeLa and C33A cells during serum starvation by western blot analysis. Compared to the control, the levels of CREB1 increased 2.1 and 2.4-fold in HeLa cells and 2.3 and 1.6-fold in C33A cells that were serum starved for 12 and 24 h, respectively (Figure 6B). Thus, short-term serum starvation could lead to elevated levels of miR-320a and CREB1. To demonstrate that CREB1 affects the transcription of the miR-320a promoter, a CREB1 expression plasmid (pcDNA3/HA-CREB1) and a vector to silence CREB1 (pSilencer/CREB1) that we generated previously were transfected into HeLa and C33A cells to test their effectiveness by western blot analysis. pcDNA3/HA-CREB1 led to a 2.5 and 2.1-fold increase in CREB1 expression, and pSilencer/CREB1 resulted in a 50% and 60% reduction in CREB1 expression (Figure 6C).

First, we transiently overexpressed CREB1 and a control vector in HeLa and C33A cervical cancer cells. Then, upon examining the endogenous levels of miR-320a, we found that miR-320a increased by 2.1 and 1.7-fold, whereas CREB1 knockdown decreased the levels of miR-320a by approximately 40% and 30% (Figure 6D). Then, we transiently overexpressed CREB1 and a control vector together with the promoter of miR-320a (miR-320a-p1180) in HeLa and C33A cells. CREB1 overexpression increased luciferase activity by approximately 2.3 and 1.6-fold, whereas shR-CREB1 reduced luciferase activity by approximately 30% and 25%, respectively (Figure 6E).

To further determine whether CREB1 directly binds to the promoter of miR-320a to activate its expression, three constructs with mutations in the CREB1 binding sites in the promoter of miR-320a-1180 were generated: pGL3/luc-miR320a-p1180-103-CREB1-mut (single mutant binding site at 103), pGL3/luc-miR320a-p1180-614-CREB1-mut (single mutant binding site at 614) and pGL3/luc-miR320a-p1180-103-614-CREB1-mut (double mutant binding sites at 103 and 614). All three are deletion mutations (Figure 6A). Then, pcDNA3/HA-CREB1 and the miR-320a promoter mutants were co-transfected into HeLa and C33A cells. Luciferase activity analysis showed that both single mutations lead to reduced CREB1 activation of the miR-320a-p1180 promoter and that the double mutant almost abolished the activation of CREB1 on the miR-320a-p1180 promoter (Figure 6F and 6G). To further explore whether CREB1 could directly target the miR-320a promoter, we performed a chromatin immunoprecipitation (ChIP) assay in HeLa cells, which demonstrated that CREB1 binds directly to the sites at 103 and 614 of the miR-320a promoter (Figure 6H and 6I). Altogether, these data indicated that CREB1 binds to the promoter of miR-320a to activate its expression.

In general, cancer cells are relatively serum starved, and CREB1 is upregulated in cancer tissues [30]. Our findings indicate that CREB1 activates the transcription of miR-320a. To investigate whether this phenomenon is common among cervical cancers, we detected the expression levels of CREB1 and miR-320a in ten paired cervical cancer tissues and adjacent non-tumor tissues by qRT-PCR. The data showed that both CREB1 and miR-320a were upregulated in cervical cancer tissues compared to adjacent non-tumor tissues in eight of the ten specimens (Figure 6J and 6K).

## DISCUSSION

Recently, increasing evidence has suggested that miRNAs play important roles in mediating human responses to stresses, such as ultraviolet rays, ischemia, hypoxia, inflammation, electric shock, and starvation [31]. For tumors, quick expansion leads to an ischemic environment. Additionally, serum starvation could lead to cell death. Thus, the theory of “tumor starvation therapy” aims at blocking the blood supply to tumors using anti-tumor drugs [32]. However, serum starvation was recently shown to lead to alterations in miRNA expression profiles, and miR-17 could allow glioblastoma cells to survive longer during serum starvation and could target MDM2 and PTEN with MDM2 acting as an oncogene [6]. A previous study has also reported that miR-320a is associated with environmental stress; this miR is induced under oxidative stress and regulates glycolysis [33]. Our study focused on other aspects of miR-320a during environmental stress. miR-320a was screened and shown to be upregulated in HeLa and C33A cells during serum starvation. We also found that miR-320a expression could prolong cervical cancer cell survival by improving mitophagy during the stress of serum starvation, which is opposite to the “tumor starvation therapy” function of miR-17. To determine how miR-320a exerted its effects, we identified the core promoter region and binding sites of the transcription factor CREB1. Recently, Ao et al. discovered that E2A could control the cell cycle by targeting the promoter sequence of miR-320a [27], although their results would be more convincing if they had generated the promoter region of miR-320a and examined how E2A regulates miR-320a expression. Transcription factors are powerful tools for miRNA regulation. The Zinc-finger transcription factors Runt-related transcription factor-2 (Runx2) [34], p53 [35], sex-determining region Y box 9 (SOX9) [36], GATA-binding protein 1 (GATA-1) [37] and CREB1 are all well documented. CREB1 plays an important role in regulating MUC18 in the metastatic pathway of melanoma cells [38], and CREB1 overexpression correlates with acute myeloid leukemia (AML) [39]. We demonstrated that CREB1 is a positive regulator of miR-320a.

Cervical cancer is a common cancer in females. Although the introduction of cervical cytological scanning and improved early diagnosis have significantly decreased the morbidity and mortality of this disease in the past four decades [40, 41], the molecular mechanisms of this disease, which could contribute to biomarker development, remain unclear. miRNAs are involved in the progression of cervical cancer and function as oncogenes and tumor suppressor genes in cervical cancer [42, 43]. Studies regarding miRNAs in the cervical cancer cell lines are meaningful for improving the recovery rate and have great potential for being applied to future clinical therapy.

It has been well established that serum starvation induces autophagy. We have proven that at least part of the autophagy process occurs in mitochondria. Mitochondrial autophagy, or mitophagy, is a major mechanism involved in mitochondrial quality control by selectively removing damaged or unwanted mitochondria. Mitochondria play a central role in the production of cellular ATP and metabolites that are required for normal cellular activities and a role in programmed cell death [44]. Starvation causes the deactivation of mTOR, which then activates autophagy. miR-376a is a regulator of starvation-induced autophagy [45]. The cytokine macrophage migration inhibitory factor (MIF) plays a permissive role in the maintenance of cardiac contractile function during starvation by regulating autophagy [46]. This study is the first to report that CREB1 can bind to the promoter of miR-320a and activate its expression, which in turn affects survival through mitophagy.

To our knowledge, we are the first to report that miR-320a is a miRNA associated with cancer cell mitophagy through transcriptional regulation. Previous research has shown that miR-320a has the following different roles in different cancers: a cancer suppressor gene in colon [47] and colorectal cancer [48], an inhibitor of cell proliferation in leukemic cells [10], and an oncogene in hepatocellular carcinoma [49]. These reports have shown that the level of miR-320a varies in different types of cancer cells and that these varying levels may regulate mitophagy to exert its different roles. Therefore, the function of miR-320a in different cancers is regulated part via the control of mitophagy. Additionally, the relationship between mitophagy and cancer is complex. First, the effect of mitophagy may be different in different types of cancer cells. Second, even in the same cell, the role of mitophagy may be different under different external factors. Third, the function of mitophagy may be different during different stages of tumor development. During the early stage of tumor development, tumor tissues lack vasculature and nutrition; thus, mitophagy is enhanced. In contrast, at the developmental stage, the activity of mitophagy is reduced [50]. Therefore, our finding that the levels of miR-320a in certain cervical cancer tissues are elevated and that others are reduced may be because the individual tumors are at different stages.

VDAC1 and mitochondria are inseparable. VDAC1 resides in the outer mitochondrial membrane and forms a common pathway for the exchange of metabolites between the mitochondria and the cytosol, thus playing a crucial role in the regulation of metabolic and energetic functions. Increasing evidence has demonstrated that VDAC1 has multiple important roles. VDAC1 is a channel that transport cations such as Ca2+, as well as many charged and non-charged metabolites [51]. Accumulating evidence also points to VDAC as a key player in the regulation of mitochondria-mediated apoptosis and in cancers and neurodegenerative disorders [52]. The importance of VDAC1 in cancer cells is further reflected in the finding that silencing VDAC1 expression reduces cellular ATP levels and cellular growth [53]. Furthermore, when HeLa cervical cancer cells stably expressing shRNA directed against VDAC1 were injected into nude mice, the development of a solid tumor was inhibited [54]. We focused on the decreased expression of VDAC1 and mitophagy. In fact, an association between VDAC1 and mitophagy has been previously reported primarily in Parkinson's disease [28]. We confirmed this association in cervical cancer cell lines. However, Jian Li et al [55] reported that voltage-dependent anion channels (VDACs) promote mitophagy to protect neuron from death in an early brain injury following a subarachnoid hemorrhage (SAH) in rats. Mitophagy plays an important protective role at 48 h, a key time point of early brain injury following SAH, and that VDAC helps elicit the molecular pathway of mitophagy. When the voltage-dependent anion channels were inhibited by VDAC1 siRNA, the expression of microtubule-associated protein 1 light chain 3 was attenuated. Because VDAC1 closely correlates with apoptosis and is a target of miR-320a, miR-320a could be a potential therapeutic target. Further research will be performed to clarify the relationship between miR-320a and apoptosis in subsequent studies.

**Figure 7:**
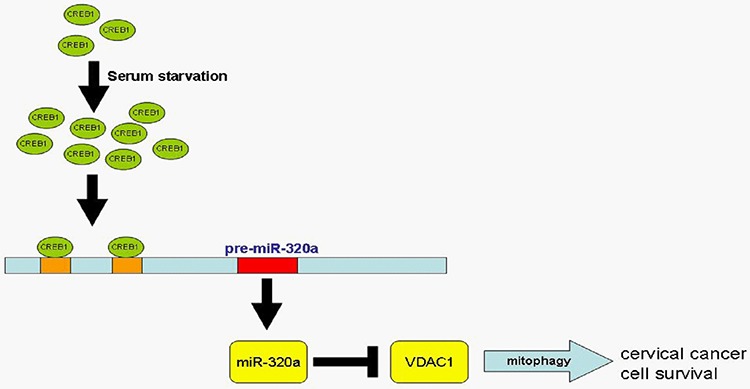
Pathway of CREB1-driven expression of miR-320a-induced mitophagy In response to serum starvation, miR-320a expression is enhanced because of CREB1 activation, and the upregulation of miR-320a directly targets and represses VDAC1 expression, which induces mitophagy and contributes to the survival of cervical cancer cells.

In summary, we studied miR-320a at both the transcriptional and post-transcriptional levels. We generated the promoter of miR-320a and, more importantly, found that the core promoter is located in the upstream −16 to −130 region of pre-miR-320a. We present convincing evidence showing that serum starvation and CREB1 could increase the transcription of miR-320a, that serum starvation improves the proliferation of HeLa and C33A cervical cancer cells through miR-320a, that miR-320a prolonged the survival of cervical cancer cells, and that repressing the expression of VDAC1 enhanced mitophagy. We thus proposed a signaling pathway delineating miR-320a activities (Figure 7). This finding reveals a novel mechanism for the stress response in human cervical cancer cells and provides new insight into tumor development.

## MATERIALS AND METHODS

### Clinical human cervical carcinoma specimens and RNA isolation

Ten pairs of human cervical carcinoma tissues that consisted of cervical carcinoma tissue and adjacent non-tumorous cervical tissue were collected at the Cancer Center of Sun Yat-Sen University of Medical Sciences. Informed consent was obtained from each patient, and ethics approval was granted by the Ethics Committee of Sun Yat-Sen University of Medical Sciences. RNA was isolated from the tissue samples using a mirVana miRNA Isolation Kit (Ambion, Austin, TX, USA) according to the manufacturer's instructions.

### miRNA microarray analysis

The technique of cloning and amplifying microRNAs was performed by microRNA labeling using the mirVana miRNA probe set (Ambion). Probes were resuspended at 50 mM in 3 × saline sodium citrate (SSC) and spotted on MICROMAX SuperChip I glass slides (PerkinElmer, MA, USA) in duplicate at 50%–60% humidity using a SpotArray 24 Microarray Printing System (PerkinElmer). RNAs of 18–26 nt in length were retrieved and purified from 30 μg RNA, which was fractionated on an 8M urea denaturing 15% polyacrylamide gel. Then, T4 RNA ligase (Fermentas) was used to ligate 5′ and 3′ adaptors to the retrieved RNA. A cDNA library was generated by reverse transcription using M-MLV (Promega, Madison, WI), which was reversed complementary to the 5′ adaptor. The product was amplified using asymmetric PCR to label the control group with Cy3 and the experimental group with Cy5. Hybridization buffer (Ambion) was added to the PCR product. Following heating at 95°C for 3 min, the solution was added to the slide, which contained 243 human mature miRNA-associated probes, with each probe added in triplicate. After the slides were hybridized at 42°C overnight (12–16 h), they were washed in SSC and scanned with a ScanArray Express Microarray Scanner using ScanArray 3.0 software (PerkinElmer). All of the oligonucleotide sequences were purchased from Integrated DNA Technology (IDT).

### Analysis of data obtained from microarrays

The scanned data were analyzed using ScanArray Express version 1.0. The intensity of the spots was extracted and normalized, and the signal intensity minus the background was regarded as the expression level. Spots with expression levels lower than 300 (for miRNA microarray) or 1000 (for cDNA microarray) were excluded. In the miRNA microarray, we selected miRNAs with expression levels between cancer tissue samples and matched normal tissue samples that differed by at least 1.5-fold. In the cDNA microarray, the threshold was 2.0-fold.

### Vector construction

A cDNA sequence containing one pre-miR-320a unit was inserted into a pcDNA3 mammalian expression vector in the BamHI and EcoRI enzyme sites (Promega, Madison, WI). The sequence of pre-miR-320a is identical to the endogenous sequence. The promoter fragments of miR-320a were cloned by PCR, and the PCR products were cloned into the pGL3-Basic/luciferase reporter vector between the HindIII and NheI sites. The generated plasmids were as follows: pGL3-luc-miR-320a-p1180bp (Pro-320a-1205-S and Pro-320a-25-AS primers), pGL3-luc-miR-320a-p722bp (Pro-320a-1205-S and Pro-320a-484-AS primers), pGL3-luc-miR-320a-p497bp (Pro-320a-503-S and Pro-320a-25-AS primers), pGL3-luc-miR-320a-p408bp (Pro-320a-89-S and Pro-320a-89-406-AS primers), pGL3-luc-miR-320a-p308bp (Pro-320a-308-S and Pro-320a-25-AS primers), pGL3-luc-miR-320a-p202bp (Pro-320a-202-S and Pro-320a-25-AS primers), and pGL3-luc-miR-320a-p89bp (Pro-320a-89-S and Pro-320a-25-AS primers). The generation of CREB1 shRNA and cDNA plasmids has been described in our previous work.

The 3′ UTR segment of the VDAC1 gene carrying the predicted miR-320a binding site was amplified by PCR. The PCR products were cloned into the pcDNA3/EGFP vector between the BamHI and EcoRI sites; the mutant segment was generated by PCR site-directed mutagenesis. Then, the product was inserted into pcDNA3/EGFP using the same enzyme sites.

Two CREB1 binding sites are present in the promoter of miR-320a. To generate mutants containing the single and double mutations of the CREB1 target sites, we first generated the single mutation pGL3/luc-miR320a-p1180-103-CREB1-mut (single mutant binding site at 103) and pGL3/luc-miR320a-p1180-614-CREB1-mut (single mutant binding site at 614). Then, we used pGL3/luc-miR320a-1180-103-CREB1-mut, with a mutation in the single binding site at 614, to generate the double mutant pGL3/luc-miR320a-p1180-103-614-CREB1-mut. All of these alterations are deletion mutations (Figure 6A). The inserts were confirmed by DNA sequencing. All of the primers used are listed in Table 1.

**Table 1:** The primers and oligonucleotides used in this work

Name	Sequence
Pri-miR-320a-S	5′-GTTGGATCCGGCGTTTCCTTCCGACATG-3′
Pri-miR-320a-AS	5′-GCTGAATTCGTCCACTGCGGCTGTTCC-3′
Pro-320a-1205-S	5′-CCAGCTAGCGACAACGGCGAGACTTCCTCTC-3′
Pro-320a-503-S	5′-GATGCTAGCGTTTCCTTCCGACATGTTGC-3′
Pro-320a-308-S	5′-GACGCTAGCACCCAGGTGAGAGCCTTTG-3′
Pro-320a-202-S	5′-GATGCTAGCTCACCTGCAACGCGACC-3′
Pro-320a-89-S	5′-GATGCTAGCCGGGACTGGGCCACAG-3′
Pro-320a-25-AS	5′-CTCAAGCTTACCCAGCTTTTCCCGACTC-3′
Pro-320a-484-AS	5′-GCGAAGCTTGCAACATGTCGGAAGGAAAC-3′
Pro-320a-89-406-AS	5′-CTCAAGCTTCTGTGGCCCAGTCCCGC-3′
ASO-miR-320a	5′-TCGCCCTCTCAACCCAGCTTTT-3′
ASO-NC	5′-UGACUGUACUGAGACUCGACUG-3′
VDAC1-3′ UTR-S	5′-GGCGGATCCATGTCTGGGATGCAAGTA-3′
VDAC1-3′ UTR-AS	5′-GGCGGCGAATTCATCAATTAGGGTTAGGGA-3′
VDAC1-3′ UTR-MS	5′-GTTGATTCGTGTATAAGATGT-3′
VDAC1-3′ UTR-MA	5′-ACATCTTATACACGAATCAAC-3′
CREB1-pro-320a-614-MS	5′-CTTCTTGACTCCCCCCCTGGAACGGATGGAG-3′
CREB1-pro-320a-614-MAS	5′-CCATCCGTTCCAGGGGGGGAGTCAAGAA GGTAC-3′
CREB1-pro-320a-103-MS	5′-GGCGCGGGGCGGAAGGGGGGCGGGACTGGG-3′
CREB1-pro-320a-103-MAS	5′-CCAGTCCCGCCCCCCTTCCGCCCCGCGCCAAG-3′
miR-320a RT primer	5′-TCGTATCCAGTGCAGGGTCCGAGGTGCACTGGATACG ACTCGCCCTC-3′
U6 RT primer	5′-GTCGTATCCAGTGCAGGGTCCGAGGTGCACTGGATACG ACAAAATATGG-3′
miR-320a forward primer	5′-TGCGGAAAAGCTGGGTTGAGAGG-3′
U6 forward primer	5′-TGCGGGTGCTCGCTTCGGCAGC-3′
U6 reverse primer	5′-CCAGTGCAGGGTCCGAGGT-3′
VDAC1-qPCR-S	5′-TGACGCCTGCTTCTCG-3′
VDAC1-qPCR-AS	5′-GCCACCAAGTTCTCCC-3′
CREB1-qPCR-S	5′-TTTCTCCTCCCACCGCC-3′
CREB1-qPCR-AS	5′-GTACACGAACATTCATAACAGC-3′
β-Actin-S	5′-CGTGACATTAAGGAGAAGCTG-3′
β-Actin-AS	5′-CTAGAAGCATTTGCGGTGGAC-3′
p320a-CREB1-chip-S1	5′-TTGAATCCTGGGGCTTGG-3′
p320a-CREB1-chip-AS1	5′-CCGACCTCTCCTGACTGG-3′
p320a-CREB1-chip-S2	5′-CCAGCCGCCAGCCTTCGGTCTC-3′
p320a-CREB1-chip-AS2	5′-TCGCCCTCTCAACCCAGC-3′
5′ adaptor	5′-CTGTAGGCACCATCAAx-3′ (x:DMT-OC3-CPG)
3′ adaptor	5′-ACTCGAGAAUUCCGAAA-3′
cDNA library RT primer	5′-TTGATGGTGCCTACAG-3′
Asymmetric PCR sense primer	5′-ACTCGAGAATTCCGAAA-3′
Asymmetric PCR antisense primer	5′-Cy3/Cy5-CACTTGATGGTGCCTACAG-3′

### Cell culture, starvation, transfection, and RNA extraction

The human cervical cancer HeLa and C33A cell lines were cultured in RPMI 1640 and MEM-α medium (Gibco), respectively. The culture medium was supplemented with 10% fetal bovine serum (FBS) (MinHai Bio-Engineering, China), 100 IU/mL of penicillin, 100 μg/mL of streptomycin and 2 mM glutamine in a humidified atmosphere at 37°C with 5% CO_2_. Cells were washed three times with PBS solution before starvation and then cultured in RPMI 1640 or MEM-α medium without FBS. The HeLa or C33A cells were transfected using Lipofectamine™ 2000 transfection reagent (Invitrogen, Carlsbad, CA) in antibiotic-free Opti-MEM medium (Invitrogen, Carlsbad, CA) following the manufacturer's instructions. Total RNA was extracted using TRIzol reagent (Invitrogen, Carlsbad, CA), and miRNAs were obtained using a mirVana miRNA Isolation Kit (Ambion, Austin, TX). Each experiment in this study was performed at least three times.

### qRT-PCR

To detect the relative level of mRNAs or mature miRNAs by qRT-PCR, we used SYBR Premix Ex Taq™ (TaKaRa, Otsu, and Shiga, Japan) according to the manufacturer's protocols. All PCR experiments were performed under the following conditions: 94°C for 4 min, followed by 40 cycles of 94°C for 30 s, 58°C for 30 s and 72°C for 30 s in an iQ5 real-time PCR system (Bio-Rad). The real-time PCR results were analyzed and expressed as relative miRNA expression of the CT (cycle threshold) value using the 2^−ΔΔCT^ method. The primers for human U6 RNA were used as real-time PCR controls (Table 1).

### Western blot

Cell lysates were prepared and extracted at 48 h post-transfection using RIPA buffer (10 mM Tris-HCl (pH 7.4), 1% Triton X-100, 0.1% SDS, 1% NP-40, and 1 mM MgCl_2_). Protein expression was analyzed by western blot. The proteins were transferred onto a PVDF membrane. After the protein bands were detected, the blot was re-probed with anti-GAPDH antibody to confirm equal loading of the samples. Band intensity was quantified using Lab Works 4.0 (UVP, Upland, CA, USA). The following antibodies were used: rabbit anti-CREB1 (1:1000), mouse anti-LC3 (1:1000), rabbit anti-GAPDH (1:2000), goat anti-mouse (1:2500) and goat anti-rabbit (1:2500) (Tianjin Saier Biotech, China).

### Cell proliferation assay

To determine relative cell proliferation, MTT assays were performed. HeLa and C33A cells were seeded in 96-well plates. The absorbance at 570 nm was detected using a μQuant Universal Microplate Spectrophotometer (Bio-Rad, Hercules, CA).

### Luciferase assay

HeLa and C33A cells were seeded in 48-well plates at a density of 1.0 × 10^4^ cells per well in RPMI 1640 and MEM-α medium containing 10% FBS one day before transfection. The cultures were maintained at 37°C for 24 h, followed by co-transfection with the luciferase reporter constructs and CREB1 using Lipofectamine™ 2000 as described in the manufacturer's protocols. Then, the cells were collected and lysed after 24 h using a Dual-Luciferase Reporter Assay System kit (Promega, America). The *Renilla* luciferase expression vector pRL-TK was used as the internal control. The intensities of firefly and *Renilla* luciferase were detected using a Modulus™ single tube multimode reader (Turner Biosystems, America). All of the experiments were repeated three times.

### GFP-LC3 dot assay and mitophagy

A green fluorescent protein (GFP)-tagged LC3 (GFP-LC3) expression plasmid was generated. LC3-I is cytosolic; after LC3-I is processed into LC3-II, the latter is associated with the autophagosome membrane. GFP-LC3 dots can be quantified by either the number of dots per cell or the number of cells with GFP-LC3 dots exceeding the average number of dots in the control cells. HeLa cervical cancer cells were transiently transfected with the GFP-LC3 vector using Lipofectamine™ 2000 (Invitrogen, Carlsbad, CA) in antibiotic-free Opti-MEM medium (Invitrogen, Carlsbad, CA) following the manufacturer's instructions. The cells were examined under a fluorescence microscope. We counted the number of autophagic cells showing GFP-LC3 dots (≥5 dots/cell) among 200 GFP-positive cells.

Mitophagy was induced using 6-OHDA (Sigma) 24 h before serum starvation or after transfection. The cells were then stained for 15 min with 100 nM MitoTracker Red (Sigma, America) (diluted in FBS-free RPMI 1640 medium) in a humidified atmosphere at 37°C with 5% CO_2_ and kept in a dark place before testing for mitophagy.

### ChIP assays

ChIP assays were performed using an EZ-ChIP™ Chromatin Immunoprecipitation Kit (Millipore, Billerica, MA, USA) according to the manufacturer's instructions. HeLa cells were seeded in 10 cm cell culture plates. Then, the cells were lysed, sonicated to shear DNA and immunoprecipitated with anti-CREB1 (Tianjin Saier Biotech, China) or control antibodies (IgG and GAPDH). qPCR primers were designed using PrimerBLAST (Table 1), and qPCR was performed to purify the DNA CREB1/DNA crosslink.

### Statistical analysis

The values of all figures are presented as the mean ± standard deviation (SD). The statistical analyses for the data comparisons were performed using a paired *t*-test. *p* < 0.05 was considered statistically significant (*p < 0.05, **p < 0.01, ***p < 0.001).
